# Associations between sleep duration trajectories and physical dysfunction among middle-aged and older Chinese adults

**DOI:** 10.1186/s12889-025-23870-2

**Published:** 2025-07-30

**Authors:** Xiaojiang Zhao, Laiguo Han, Hong Ding, Changqing Li

**Affiliations:** Department of Physical Education and Arts, Bengbu Medical University, Bengbu, China

**Keywords:** Sleep duration trajectory, Physical dysfunction, Group-based trajectory models, CHARLS

## Abstract

**Background:**

The relationship between a single time-point measurement of sleep duration and physical dysfunction has been extensively investigated. However, few researches has concentrated on the effects of sleep duration trajectories. This study sought to evaluate the association between sleep duration trajectories and physical dysfunction in a longitudinal cohort of middle-aged and older Chinese individuals.

**Methods:**

This research included a large pool of subjects (*n* = 7157) between the ages of 45 and 80 from the China Longitudinal Study of Health and Retirement (CHARLS). Utilizing sleep duration data collected periodically between 2011 and 2015, the sleep duration trajectory was plotted using the group-based trajectory modeling (GBTM). Physical dysfunction was evaluated using data from 2015. Multivariable logistic regression model was then used to examine the risk of physical dysfunction with different sleep time trajectories.

**Results:**

Three distinct sleep duration trajectories were identified: class 1, consistently long sleep duration(*n* = 2504, 34.98%); Class 2: consistently moderate sleep duration(*n* = 2338, 32.67%); Class 3: consistently short sleep duration( *n* = 2315, 32.35%). Multivariable logistic regression revealed that compared with consistently moderate sleep duration, consistently short sleep duration was significantly positively correlated with the risk of physical dysfunction in unadjusted model and adjusted model (OR: 1.75, 95% CI: 1.54 ~ 1.99; *p* < 0.001).

**Conclusions:**

Consistently short sleep duration trajectories are positively correlated with physical dysfunction compared to participants with consistently moderate sleep duration trajectories. The study points out the significant importance of keeping an eye on how sleep duration changes over time.

**Supplementary Information:**

The online version contains supplementary material available at 10.1186/s12889-025-23870-2.

## Introduction

Physical function refers to the process of converting physiological signals into muscle movements, like walking, balancing, and standing [1]. The maintenance of physical function in middle-aged and older adults is crucial for independent living, quality of life, and longevity [2–4]. However, as individuals age, there is a progressive decline in muscle strength, postural balance, and aerobic capacity, leading to physical dysfunction. Numerous studies have demonstrated that physical dysfunction has emerged as a prevalent health issue among middle-aged and elderly populations [5]. Prior investigations have shown a significant link between physical dysfunction and decreased physical health, lower quality of life, and heightened disability [6, 7]. According to related data, by the year 2020, the elderly population in China experiencing physical dysfunction had reached 43.75 million, with projections indicating an increase to over 90 million by 2050 [8]. The significant adverse effects associated with physical dysfunction have imposed substantial economic and social burdens on society, thereby emerging as a major public health challenge [9]. Recognizing adjustable lifestyle risk factors is vital for preventing and managing physical dysfunction in middle-aged and elderly individuals. Recent research has underscored the significance of sleep duration in the onset of physical dysfunction [10, 11]. Nevertheless, most studies assess sleep duration at one time point, longitudinal patterns have not been adequately investigated.

Sleep is a behavior that can be modified, improving sleep can improve other health behaviors [12]. Along with many other physiological changes, a person's sleep patterns evolve over time [13]. More and more studies have shown that the sleep time of middle-aged and elderly people is related to various health problems, and proper sleep is crucial to regulate metabolic and physiological functions. Insufficient and excessive sleep has been associated with several age-related disorders, including sarcopenia, frailty, and metabolic syndrome [14, 15]. For example, one meta-analysis shows that sleep is u-shaped relationship between time and reduce muscle disease, suggests that both insufficient and excessive sleep may negatively affect physical function [16]. In the Japanese cohort, individuals who slept longer were more likely to develop sarcopenia or decreased physical function [17, 18]. Nevertheless, the aforementioned study employed only a singular measurement of sleep duration, neglecting the variability of sleep duration across different age groups. Consequently, relying on a single measurement may not accurately capture the relationship between sleep duration and physical dysfunction. Furthermore, while some evidence indicates that a consistently short sleep duration is linked to various adverse health outcomes in comparison to a normal sleep duration, the association between sleep duration trajectory and physical dysfunction remains unreported.

In view of this, we conducted a longitudinal evaluation of sleep duration within the China Health and Retirement Longitudinal Study (CHARLS) to explore the relationship between changes in sleep duration trajectories over four years and the likelihood of physical dysfunction. The findings of this study may provide valuable insights into the role of detecting changes in sleep trajectories in preventing physical dysfunction.

## Methods

### Study population

The data for the current study were derived from the CHARLS, a comprehensive nationwide prospective cohort study. This study encompasses health information and economic status data for adults aged 45 and above across 28 provinces in China. CHARLS utilized a multistage stratified probability proportional-to-size sampling methodology to recruit participants from both rural and urban areas, encompassing 150 counties or districts within the 28 provinces. The national baseline survey was conducted in 2011. Subsequently, all participants were subject to follow-up assessments every 2–3 years, with additional participants being recruited over time. Previous literature has documented CHARLS methodologies in detail [19]. The Peking University Biomedical Ethics Review Committee (IRB00001052-11015) approved the study protocol, and all participants gave written informed consent.

**Fig. 1:**
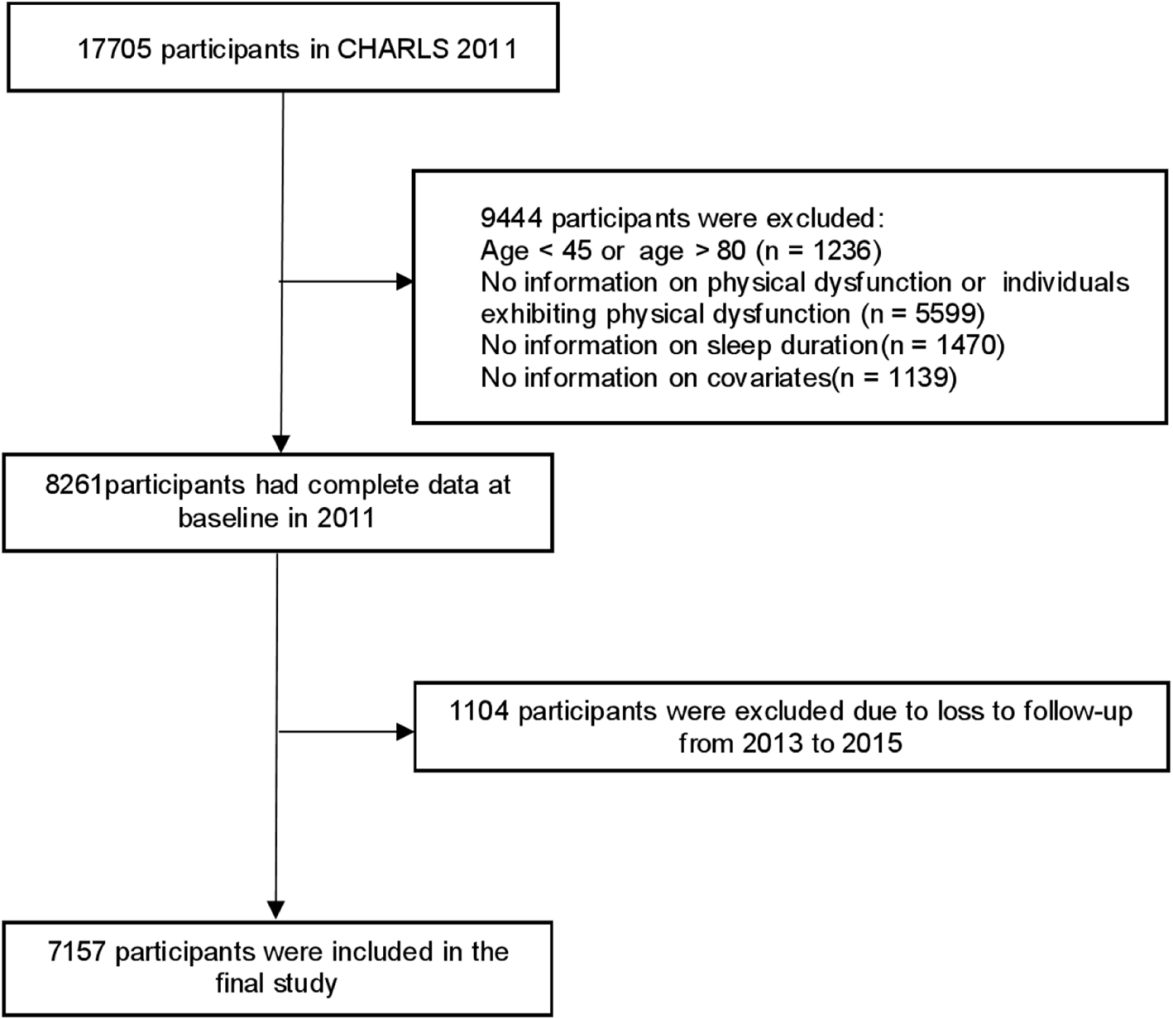
Flow diagram of participants selection. CHARLS, China Health and Retirement Longitudinal Study

There were 17,705 participants at baseline in 2011 (first wave). Design based on previous trajectory modeling studies [20]. In Wave 1, individuals were excluded if they were younger than 45 years old or older than 80 years old (*n* = 1236). Additionally, individuals were excluded for the following reasons: no information on physical dysfunction (*n* = 5599), no information on sleep duration (*n* = 1470), and no information on covariates (*n* = 1139). From the first to the third wave of follow-up, 1104 participants were lost. The final analysis included 7157 participants (Fig. 1). This study followed the Strengthening the Reporting of Observational Studies in Epidemiology (STROBE) reporting guidelines (see Supplemental Table 1 STROBE Checklist).

**Table 1:** Characteristics of all participants by sleep duration trajectory groups

Characteristics	Sleep duration trajectory				
	Total	Consistently moderate	Consistently long	Consistently short	*p* value
	*n* = 7157	*n* = 2338	*n* = 2504	*n* = 2315	
Age, Mean ± SD	57.3 ± 8.0	57.7 ± 8.2	56.6 ± 7.8	57.8 ± 8.1	< 0.001
Gender, n (%)					< 0.001
Female	3434 (48.0)	1165 (49.8)	1093 (43.7)	1176 (50.8)	
Male	3723 (52.0)	1173 (50.2)	1411 (56.3)	1139 (49.2)	
Education level, n (%)					< 0.001
High school and above	989 (13.8)	268 (11.5)	459 (18.3)	262 (11.3)	
Middle school	1797 (25.1)	542 (23.2)	728 (29.1)	527 (22.8)	
Primary school	1763 (24.6)	576 (24.6)	632 (25.2)	555 (24)	
Illiterate	2608 (36.4)	952 (40.7)	685 (27.4)	971 (41.9)	
Marital status, n (%)					0.001
Married	6553 (91.6)	2144 (91.7)	2326 (92.9)	2083 (90)	
Single	604 (8.4)	194 (8.3)	178 (7.1)	232 (10)	
Residence, n (%)					< 0.001
Rural	4449 (62.2)	1544 (66)	1438 (57.4)	1467 (63.4)	
Urban	2708 (37.8)	794 (34)	1066 (42.6)	848 (36.6)	
Smoking status, n (%)					0.002
No	3763 (52.6)	1266 (54.1)	1245 (49.7)	1252 (54.1)	
Yes	3394 (47.4)	1072 (45.9)	1259 (50.3)	1063 (45.9)	
Drinking status, n (%)					0.005
No	3739 (52.2)	1276 (54.6)	1249 (49.9)	1214 (52.4)	
Yes	3418 (47.8)	1062 (45.4)	1255 (50.1)	1101 (47.6)	
BMI, n (%)					< 0.001
Normal	3847 (53.8)	1231 (52.7)	1331 (53.2)	1285 (55.5)	
Obesity	898 (12.5)	271 (11.6)	337 (13.5)	290 (12.5)	
Overweight	338 (4.7)	119 (5.1)	121 (4.8)	98 (4.2)	
Underweight	1754 (24.5)	624 (26.7)	621 (24.8)	509 (22)	
Health status, n (%)					< 0.001
Fair	4541 (63.4)	1490 (63.7)	1582 (63.2)	1469 (63.5)	
Good	1523 (21.3)	543 (23.2)	616 (24.6)	364 (15.7)	
Poor	1093 (15.3)	305 (13)	306 (12.2)	482 (20.8)	
Physical activity levels	*n* = 7157	*n* = 2338	*n* = 2504	*n* = 2315	0.026
LPA	2219 (31.0)	760 (32.5)	754 (30.1)	705 (30.5)	
MPA	995 (13.9)	278(11.9)	408 (16.3)	309(13.3)	
HPA	3943 (55.1)	1300 (55.6)	1342 (53.6)	1301(56.2)	
Depression					< 0.001
No	4806 (67.2)	1844 (73.6)	1755 (75.1)	1207 (52.1)	
Yes	2351 (32.8)	660 (26.4)	583 (24.9)	1108 (47.9)	
Physical dysfunction, n (%)					< 0.001
Yes	2313 (32.3)	554 (23.7)	881 (35.2)	878 (37.6)	
No	4844 (67.7)	1784 (66.3)	1623 (64.8)	1437(62.4)	

Abbreviations: *BMI* Body mass index, *LPA* Low-intensity physical activity, *MPA* moderate-intensity physical activity, *HPA* High-intensity physical activity

### Assessment of physical dysfunction and sleep duration

#### Sleep duration

Consistent with prior research [21], we measured 24-h sleep duration by combining nighttime sleep and daytime naps, assessed with the questions:"How many hours per night did you typically sleep over the past month?"and"Last month, how long were your naps after lunch?"The validity of self-reported nighttime sleep duration and daytime napping has been established within the field of sleep epidemiology [22–24]. We assessed sleep duration trajectories based on data collected in 2011, 2013, and 2015.

#### Physical dysfunction

The CHARLS questionnaire includes several items related to physical function, including: the ability to run or jog 1 km, walk 1 km, walk 100 m, rise from a seated position after sitting for an extended period, ascend multiple flights of stairs consecutively, bend over, bend knees or squat, stretch arms overhead, and pick up a coin. Responses are organized into four tiers: (1) No trouble; (2) Some trouble; (3) Need assistance; (4) Unable to do. Referring to the previous research [25, 26]. Responses indicating"difficulty but can still be completed,""difficulty and requires assistance,"or"unable to complete"are categorized as experiencing difficulty. If a subject reported difficulty with any of the these items, they were defined as having a physical dysfunction.

#### Covariates

The covariates of the study encompassed demographic factors(age, sex, residential area, education level, cohabitation status), health status (body mass index (BMI), comorbidities, visual, hearing impairments and depression), and health behaviors (smoking, drinking and physical activity levels) at baseline. Residential areas were categorized as rural or urban; education levels as illiterate, primary/middle school, or high school and above. Cohabitation status was grouped into cohabiting or living alone. Comorbidities included conditions like hypertension, diabetes, cancer, and others. Hypertension was defined as use of antihypertensive drugs, including traditional Chinese herbal products. Respondents self-reported other comorbidities, which were categorized as 0, 1, or ≥ 2. Visual and hearing impairments were self-reported as either present or absent. The CHARLS survey uses the CES-D-10 scale to evaluate depressive symptoms in older adults. This 10-item scale includes eight negative and two positive emotion assessments, scored from 0 to 3. A total score of 10 or more indicates depression, while a score below 10 indicates no depression [27]. Smoking and drinking statuses were classified as never, current, or former smoker/drinker. The physical activity levels of respondents were evaluated utilizing data derived from the"Lifestyle"and"Health Behavior"sections of the CHARLS questionnaire. According to prior studies [28], physical activity levels were performed.Initially, respondents were prompted to review and report the duration of time they dedicated to various forms of physical activity during the preceding week. The frequency of physical activity was subsequently classified into four distinct levels. Furthermore, each type of physical activity was assigned a corresponding Metabolic Equivalent (MET) value to quantitatively assess the intensity of the activities, with higher MET values indicating greater levels of physical activity. Ultimately, based on the total MET minutes accumulated per week, respondents'physical activity levels were categorized into three groups: high-intensity physical activity (≥ 3,000 METs/week), moderate-intensity physical activity (600–3,000 METs/week), and low-intensity physical activity (≤ 600 METs/week).

### Statistical analysis

Analyses were performed with R 4.3.3 and Free Statistics software 2.0. Initially, we controlled for age, sex, and education, to derive predicted sleep duration. Subsequently, we employed the following formula to compute the adjusted Z scores: Z = (Y -Y')/RMSE, where Y is the original sleep duration, Y'indicates the estimated average score of the population, and RMSE refers to the root mean square error linked to the regression formula [20, 29, 30]. Converted Z-scores are used for analysis. We employed group-based trajectory modeling (GBTM) to determine unique patterns of sleep duration based on the current age at each visit. Missing sleep duration measures were assumed to be missing at random, allowing all available data to be incorporated into model estimation. Continuous sleep duration Z-scores were modeled as censored normal distributions [31]. The GBTM involved identifying the optimal number and shape of latent trajectories by fitting models with up to six classes. Each trajectory was assessed using linear, quadratic, or cubic growth parameters to determine the best-fitting polynomial for changes in sleep duration. Model selection was based on the Akaike information criterion (AIC) and Bayesian information criterion (BIC), with lower absolute values indicating better fit. Trajectory classification accuracy was confirmed by an average posterior probability (APP) of ≥ 70% for individual assignments. To ensure robustness in subsequent analyses, only models with > 5% of participants in each trajectory group were retained. After identifying distinct sleep duration trajectories, chi-square tests and analysis of variance (ANOVA) were performed to examine differences in individual characteristics across trajectory groups. Associations between sleep duration trajectories and physical dysfunction were then assessed using a multinomial logistic regression model, with results reported as odds ratios (OR) and 95% confidence intervals (CI). The model was adjusted for all covariates. Subgroup analyses were conducted, stratified by age (< 65, ≥ 65 years), sex, residence, educational attainment, marital status, smoking status, drinking status, BMI, health status, depression, and physical activity levels, to assess the association between trajectories of sleep duration and physical dysfunction. Statistical significance was determined by a two-sided *p*-value being below 0.05.

## Results

### Sleep duration trajectories and baseline characteristics

**Fig. 2:**
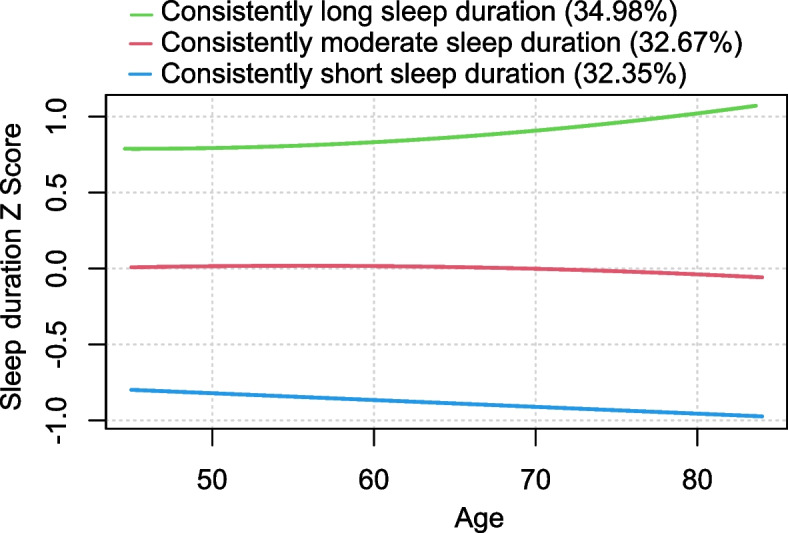
Trajectories of sleep duration by increasing age among middle-aged and older Chinese adults

For the 7157 individuals included in the analyses, the mean age was 57.3 years with a standard deviation of 8.0 years, and 51.89% of the participants were male. Descriptive statistics for sleep duration at each survey wave, along with baseline covariates, are presented in supplementary Table 2. Model goodness-of-fit was evaluated using the AIC, BIC, and APP (Supplementary Table 3). Based on these metrics, the three-trajectory GBTM model was identified as optimal. Figure 2 displays the three sleep duration trajectories plotted against participants'current age at each survey wave. For interpretability, we labeled the distinct trajectories according to their modeled graphical patterns: class 1, consistently long sleep duration(*n* = 2504, 34.98%);Class 2: consistently moderate sleep duration(*n* = 2338, 32.67%); Class 3: consistently short sleep duration(*n* = 2315, 32.35%). Supplementary Table 4 presents the maximum likelihood estimates for the final three-group trajectory model, while Table 1 details participant characteristics based on sleep duration trajectories. Participants with consistently short sleep durations were more often female, older, single, rural, underweight, less educated, in poor health, and depression compared to those with consistently moderate sleep durations (*P* < 0.05). Supplementary Table 5 shows the 24-h average sleep duration, nighttime sleep duration and daytime sleep duration of different sleep duration trajectory groups.

### Longitudinal association between sleep duration trajectories and physical dysfunction

Table 2 illustrates the relationship between sleep duration trajectories and physical dysfunction. In the unadjusted model, compared with consistently moderate sleep duration, consistently short sleep duration was positively correlated with the risk of physical dysfunction (OR: 1.75, 95%CI: 1.54 ~ 1.99; *p* < 0.001). The association was still significant in adjusted model (OR: 1.70, 95%CI: 1.49 ~ 1.93; *p* < 0.001). Indicating that participants with consistently short sleep duration were more likely to have physical dysfunction. However, there was no notable association between consistently long sleep duration and physical dysfunction in unadjusted model and adjusted model. OR and 95%CI were (OR: 0.90, 95%CI: 0.80 ~ 1.01; *p* = 0.08) and (OR: 1.09, 95%CI: 0.97 ~ 1.2; *p* = 0.07).

**Table 2:** Multinomial logistic regression analysis for the relationship between sleep duration trajectory and physical dysfunction

Characteristics		Consistently long sleep duration		Consistently short sleep duration	
		OR(95% CI)	*p* value	OR(95% CI)	*p* value
Physical dysfunction	Unadjusted model				
No		1(Ref)		1(Ref)	
Yes		0.90(0.80 ~ 1.01)	0.08	1.75(1.54 ~ 1.99)	< 0.001
physical dysfunction	Adjusted model				
No		1(Ref)		1(Ref)	
Yes		1.09(0.97 ~ 1.20)	0.07	1.70(1.49 ~ 1.93)	< 0.001

Take the consistently moderate sleep duration as a reference. Adjusted for age, sex, educational level, marital status, residence, household total income, smoking status, drinking status, BMI, and health status, depression, physical activity levels

Abbreviations: *OR* Odds ratio, *95% CI* 95% Confidence interval, *BMI* Body mass index

### Subgroup analysis

To further investigate the relationship between sleep duration trajectory and the incidence of physical dysfunction, we conducted a series of subgroup analyses. As shown in Table 3, none of the subgroups significantly changed the relationship between sleep duration trajectory and physical dysfunction (P for interaction *p*-values were all > 0.05).

**Table 3:** Subgroup Analysis of sleep duration trajectory for physical dysfunction

Subgroup	Variable	OR (95%CI)	P for interaction
Age			0.92
< 65	Consistently long sleep duration	1.08 (0.91 ~ 1.29)	
	Consistently short sleep duration	1.46 (1.21 ~ 1.76)	
≧65	Consistently long sleep duration	1.03 (0.7 ~ 1.53)	
	Consistently short sleep duration	1.48 (0.99 ~ 2.22)	
Gender			0.64
Female	Consistently long sleep duration	1.17 (0.89 ~ 1.53)	
	Consistently short sleep duration	1.5 (1.13 ~ 2.00)	
Male	Consistently long sleep duration	1.01 (0.83 ~ 1.23)	
	Consistently short sleep duration	1.43 (1.15 ~ 1.76)	
Education level			0.79
High school and above	Consistently long sleep duration	1.38 (0.89 ~ 2.12)	
	Consistently short sleep duration	1.55 (0.93 ~ 2.59)	
Middle school	Consistently long sleep duration	1.07 (0.79 ~ 1.44)	
	Consistently short sleep duration	1.46 (1.05 ~ 2.02)	
Primary school	Consistently long sleep duration	1.16 (0.85 ~ 1.58)	
	Consistently short sleep duration	1.43 (1.03 ~ 1.99)	
Illiterate	Consistently long sleep duration	0.9 (0.68 ~ 1.2)	
	Consistently short sleep duration	1.42 (1.06 ~ 1.91)	
Residence			0.08
Rural	Consistently long sleep duration	1.01 (0.83 ~ 1.24)	
	Consistently short sleep duration	1.26 (1.02 ~ 1.55)	
Urban	Consistently long sleep duration	1.17 (0.9 ~ 1.53)	
	Consistently short sleep duration	1.9 (1.43 ~ 2.54)	
Marital status			0.96
Married	Consistently long sleep duration	1.07 (0.9 ~ 1.26)	
	Consistently short sleep duration	1.44 (1.2 ~ 1.71)	
Single	Consistently long sleep duration	0.99 (0.52 ~ 1.86)	
	Consistently short sleep duration	1.66 (0.86 ~ 3.2)	
Smoking status			0.45
No	Consistently long sleep duration	0.96 (0.75 ~ 1.22)	
	Consistently short sleep duration	1.51 (1.16 ~ 1.95)	
Yes	Consistently long sleep duration	1.14 (0.92 ~ 1.4)	
	Consistently short sleep duration	1.41 (1.12 ~ 1.77)	
Drinking status			0.14
No	Consistently long sleep duration	1.03 (0.8 ~ 1.32)	
	Consistently short sleep duration	1.73 (1.32 ~ 2.28)	
Yes	Consistently long sleep duration	1.09 (0.89 ~ 1.35)	
	Consistently short sleep duration	1.31 (1.05 ~ 1.62)	
BMI			0.56
Normal	Consistently long sleep duration	1.11 (0.91 ~ 1.36)	
	Consistently short sleep duration	1.58 (1.28 ~ 1.95)	
Obesity	Consistently long sleep duration	1.13 (0.53 ~ 2.38)	
	Consistently short sleep duration	1.95 (0.76 ~ 5.02)	
Overweight	Consistently long sleep duration	1.02 (0.76 ~ 1.38)	
	Consistently short sleep duration	1.24 (0.89 ~ 1.74)	
Underweight	Consistently long sleep duration	0.65 (0.27 ~ 1.58)	
	Consistently short sleep duration	0.71 (0.3 ~ 1.67)	
Health status			0.28
Fair	Consistently long sleep duration	1.16 (0.94 ~ 1.43)	
	Consistently short sleep duration	1.68 (1.34 ~ 2.11)	
Good	Consistently long sleep duration	0.91 (0.69 ~ 1.2)	
	Consistently short sleep duration	1.11 (0.82 ~ 1.51)	
Poor	Consistently long sleep duration	1.14 (0.66 ~ 1.98)	
	Consistently short sleep duration	1.40 (0.83 ~ 2.36)	
Physical activity levels	Consistently long sleep duration		0.81
LPA	Consistently short sleep duration	0.91 (0.67 ~ 1.24)	
	Consistently short sleep duration	1.21 (0.87 ~ 1.7)	
MPA	Consistently long sleep duration	0.93 (0.62 ~ 1.4)	
	Consistently short sleep duration	1.25 (0.83 ~ 1.86)	
HPA	Consistently long sleep duration	0.99 (0.83 ~ 1.18)	
	Consistently short sleep duration	1.23 (1.02 ~ 1.48)	
Depression	Consistently long sleep duration		0.44
No	Consistently short sleep duration	0.99 (0.8 ~ 1.22)	
	Consistently short sleep duration	1.13 (0.89 ~ 1.43)	
Yes	Consistently long sleep duration	0.97 (0.62 ~ 1.5)	
	Consistently short sleep duration	1.44 (0.97 ~ 2.14)	

Take the consistently moderate sleep duration as a reference. Adjusted for age, sex, educational level, marital status, residence, household total income, smoking status, drinking status, BMI, and health status

Abbreviations: *OR* Odds ratio, *95% CI* 95% confidence interval, *BMI* Body mass index, *LPA* Low-intensity physical activity, *MPA* Moderate-intensity physical activity, *HPA* high-intensity physical activity

## Discussion

This study employed a nationally representative sample, conducting a 4-year longitudinal follow-up of 7157 middle-aged and older adults in China. Three distinct sleep duration trajectories were identified: class 1: consistently long trajectory, class 2: consistently moderate trajectory, class 3: consistently short trajectory. The consistently short trajectory was significantly linked to an increased risk of physical dysfunction compared to the consistently moderate trajectory.

To our knowledge, prior studies examining the association between sleep duration and health results have largely relied on single-time point measurements, with limited consideration of longitudinal variations. In contrast, our study identified three distinct sleep duration trajectories and demonstrated their differential associations with physical dysfunction risk. Individuals in consistently short sleep duration exhibited stable short sleep duration throughout the study period, reflecting a long-term intrinsic sleep pattern that significantly differed from other trajectories. Comparisons with existing literature highlight methodological influences on trajectory classification. For example, a study of adults aged 46–83 from the Guangzhou Nutrition and Health Study (2014–2023) identified four trajectories [32]. A U.S.-based study of Black and low-SES middle-aged and older adults reported nine modeled trajectories [33]. Discrepancies in trajectory classification may arise from differences in sleep duration assessment methods. Future studies should validate these findings and investigate the mechanisms underlying trajectory formation to elucidate their clinical implications.

In our study, using the consistently moderate sleep duration trajectories as a reference group, the consistently short sleep duration trajectories group was significantly positively correlated with the risk of physical dysfunction. This indicates that consistently short sleep duration is a risk factor for physical dysfunction in middle-aged and elderly people in China. Our findings are consistent with a 4-year follow-up of 1496 men over 65 years of age with osteoporotic fractures. The study reported a link between lack of sleep and subsequent decline in physical function [34]. Another study on sleep habits and cognitive decline noted that short sleep duration (< 5 h) was associated with cognitive decline, especially in healthy individuals. While the study focused primarily on cognitive abilities, it also indirectly suggests that short sleep duration may have adverse effects on overall physical function [35]. However, in a longitudinal observational study, in which researchers followed people 65 and older for up to six years, Although long sleep duration was associated with an accelerated decline in physical function, but the short sleep duration itself did not show the same association [36]. In addition, a study that explored the effects of chronic short sleep duration on reaction time performance reported that while short sleep duration may be associated with a decline in certain cognitive functions, However, in terms of physical function, its effect is not as significant as that of long sleep duration. The variability in results observed across these studies might be attributed to their reliance on a singular evaluation of sleep length. Conversely, our findings are derived from an analysis incorporating repeated measurements of sleep duration, underscoring the significance of examining sleep duration trajectories.

Notably, our study showed that consistently long sleep duration was not associated with the risk of physical dysfunction. This finding contrasts with some reports of this association in the literature. For example, an Italian study followed for 6 years showed greater decline in physical function during long sleep durations compared to moderate and short sleep durations [35]. Another study of Korean outpatients over 65 years of age who developed atrial fibrillation and heart failure showed an association between long sleep duration and frailty [37]. A study on the relationship between long sleep duration and physical function in postmenopausal women with an average age of 61.9 years reported similar results [38]. The inconsistencies in the findings may be due to differences in the age group of the study population. This suggests that physical dysfunction caused by long sleep duration may have a cumulative effect over time. This emphasizes the importance of delineating long sleep duration.

The biological mechanisms underpinning the association between trajectories of sleep duration and physical dysfunction are intricate. Prolonged periods of chronic short sleep duration may lead to the buildup of sleep debt is linked to worsening age-related chronic diseases [39]. Given that chronic diseases significantly contribute to physical dysfunction, the association between prolonged short sleep duration and physical dysfunction becomes apparent. Sustained insufficient sleep may activate the hypothalamic–pituitary–adrenal axis, resulting in cortisol release and subsequently leading to detrimental immunological and metabolic alterations [40]. Chronic sleep deprivation is correlated with elevated levels of specific inflammatory biomarkers [41]. Furthermore, this deprivation was associated with a reduction in hippocampal volume [42], as well as elevated β-amyloid concentrations in the thalamus [43]. These alterations in physiology might cause depression and cognitive issues. Additionally, prolonged sleep deprivation could be linked to sarcopenia [44], a major contributor to physical dysfunction. In essence, lack of sleep can disrupt neuronal metabolism, and compensatory sleep might not fully repair the damage, potentially leading to long-term health problems in older adults [45].

The principal strength of this study lies in its novel identification of patterns in the temporal variations of sleep duration and their subsequent effects on the risk of physical dysfunction. By discerning high-risk groups within a substantial, population-based cohort of middle-aged and elderly adults across China, this methodology provides innovative and complementary evidence that bolsters sleep recommendations aimed at preventing physical dysfunction.Furthermore, our findings demonstrated considerable robustness to adjustments for a wide array of covariates. However, several limitations need to be considered. First, our sample comprised only middle-aged and older Chinese adults, which may limit the generalizability of the findings to other populations. Second, sleep duration was assessed via self-reports, potentially introducing measurement bias. While objective methods such as polysomnography remain impractical for large-scale population studies, self-reported sleep duration has demonstrated strong concordance with sleep diaries [46]. Notably, actigraphy itself is a validated, noninvasive objective method for sleep assessment. Future studies could leverage such tools to examine the relationship between objectively measured sleep duration trajectories and physical dysfunction. Third, reverse causality cannot be fully ruled out, as baseline impairments in physical, cognitive, or mental health—which may influence sleep duration—were not assessed. Although, people who received a diagnosis with physical dysfunction at baseline were excluded. While this study identifies an association between sleep duration trajectories and physical dysfunction, causality remains unestablished, and findings should be interpreted cautiously. Fourth, despite the inclusion of various covariates in this study, the inherent limitations of the CHARLS database precluded the consideration of certain variables, such as work schedule. Future research should aim to collect data on work schedule to further elucidate these relationships. Finally, the relatively short follow-up period precludes definitive conclusions about the long-term effects of sleep duration trajectories on physical dysfunction. Further validation with extended observation is warranted.

## Conclusions

Our study indicates that for middle-aged and older adults in China, consistently short sleep durations are positively associated with physical dysfunction compared to consistently moderate sleep durations.This underscores that consistently inadequate sleep duration poses a risk for physical impairment.To prevent physical dysfunction in middle-aged and older adults in China, it is advised to maintain moderate to long sleep durations.

## Supplementary Information

Supplementary Material 1.

## Data Availability

Original contributions from this study are available in the article and its supplementary materials. For more inquiries, please get in touch with the corresponding author.
