# Supplementary material for: Associations between sleep duration trajectories and physical dysfunction among middle-aged and older Chinese adults
Source: BMC Public Health. 2025 Jul 30;25:2600. doi: 10.1186/s12889-025-23870-2 (PMC12309035; doi:10.1186/s12889-025-23870-2)
Supplement: Supplementary file 1 — Supplementary Material 1. [file 12889_2025_23870_MOESM1_ESM.doc]

**Supplementary table 1 STROBE Statement—checklist of items that should be included in reports of observational studies.**

|  | Item No. | Recommendation | Page  No. | Relevant text from manuscript |
| --- | --- | --- | --- | --- |
| **Title and abstract** | 1 | (*a*) Indicate the study’s design with a commonly used term in the title or the abstract | 1 | Title |
|  |  | (*b*) Provide in the abstract an informative and balanced summary of what was done and what was found | 1 | Abstract |
| Introduction | | | |  |
| Background/rationale | 2 | Explain the scientific background and rationale for the investigation being reported | 1,2 | Introduction, paragraphs 1,2 |
| Objectives | 3 | State specific objectives, including any prespecified hypotheses | 2 | Introduction, paragraph 3 |
| Methods | | | |  |
| Study design | 4 | Present key elements of study design early in the paper |  | Abstract, Introduction paragraph 3 |
| Setting | 5 | Describe the setting, locations, and relevant dates, including periods of recruitment, exposure, follow-up, and data collection |  | Methods, paragraphs 1,2 |
| Participants | 6 | (*a*) *Cohort study*—Give the eligibility criteria, and the sources and methods of selection of participants. Describe methods of follow-up  *Case-control study*—Give the eligibility criteria, and the sources and methods of case ascertainment and control selection. Give the rationale for the choice of cases and controls  *Cross-sectional study*—Give the eligibility criteria, and the sources and methods of selection of participants |  | Methods, paragraphs 2 |
|  |  | (*b*) *Cohort study*—For matched studies, give matching criteria and number of exposed and unexposed  *Case-control study*—For matched studies, give matching criteria and the number of controls per case |  | Methods, paragraphs 2 |
| Variables | 7 | Clearly define all outcomes, exposures, predictors, potential confounders, and effect modifiers. Give diagnostic criteria, if applicable |  | Methods, paragraphs 3-5 |
| Data sources/ measurement | 8* | For each variable of interest, give sources of data and details of methods of assessment (measurement). Describe comparability of assessment methods if there is more than one group |  | Methods, paragraphs 3-5 |
| Bias | 9 | Describe any efforts to address potential sources of bias |  | Methods, paragraphs 6 |
| Study size | 10 | Explain how the study size was arrived at |  | Methods, paragraph 1,2 |

Continued on next page

| Quantitative variables | 11 | Explain how quantitative variables were handled in the analyses. If applicable, describe which groupings were chosen and why |  | Methods, paragraphs 6 |
| --- | --- | --- | --- | --- |
| Statistical methods | 12 | (*a*) Describe all statistical methods, including those used to control for confounding |  | Methods, paragraphs 6 |
|  |  | (*b*) Describe any methods used to examine subgroups and interactions |  | Methods, paragraphs 6 |
|  |  | (*c*) Explain how missing data were addressed |  | N/A |
|  |  | (*d*) *Cohort study*—If applicable, explain how loss to follow-up was addressed  *Case-control study*—If applicable, explain how matching of cases and controls was addressed  *Cross-sectional study*—If applicable, describe analytical methods taking account of sampling strategy |  | N/A |
|  |  | (*e*) Describe any sensitivity analyses |  | Methods, paragraphs 6 |
| Results | | | | |
| Participants | 13* | (a) Report numbers of individuals at each stage of study—eg numbers potentially eligible, examined for eligibility, confirmed eligible, included in the study, completing follow-up, and analysed |  | Methods paragraph 1,2; Results paragraph 1 |
|  |  | (b) Give reasons for non-participation at each stage |  | N/A |
|  |  | (c) Consider use of a flow diagram |  | Methods paragraph 2 |
| Descriptive data | 14* | (a) Give characteristics of study participants (eg demographic, clinical, social) and information on exposures and potential confounders |  | Results paragraph 1 |
|  |  | (b) Indicate number of participants with missing data for each variable of interest |  | N/A |
|  |  | (c) *Cohort study*—Summarise follow-up time (eg, average and total amount) |  | N/A |
| Outcome data | 15* | *Cohort study*—Report numbers of outcome events or summary measures over time |  | Results paragraph 1 |
|  |  | *Case-control study—*Report numbers in each exposure category, or summary measures of exposure |  | N/A |
|  |  | *Cross-sectional study—*Report numbers of outcome events or summary measures |  | N/A |
| Main results | 16 | (*a*) Give unadjusted estimates and, if applicable, confounder-adjusted estimates and their precision (eg, 95% confidence interval). Make clear which confounders were adjusted for and why they were included |  | Methods paragraph 6; Results paragraph 2 |
|  |  | (*b*) Report category boundaries when continuous variables were categorized |  | Results paragraph 1 |
|  |  | (*c*) If relevant, consider translating estimates of relative risk into absolute risk for a meaningful time period |  | N/A |

Continued on next page

| Other analyses | 17 | Report other analyses done—eg analyses of subgroups and interactions, and sensitivity analyses |  | Results paragraph 3 |
| --- | --- | --- | --- | --- |
| Discussion | | | | |
| Key results | 18 | Summarise key results with reference to study objectives |  | Discussion paragraphs 1 |
| Limitations | 19 | Discuss limitations of the study, taking into account sources of potential bias or imprecision. Discuss both direction and magnitude of any potential bias |  | Discussion paragraphs 6 |
| Interpretation | 20 | Give a cautious overall interpretation of results considering objectives, limitations, multiplicity of analyses, results from similar studies, and other relevant evidence |  | Discussion paragraphs 1-4 |
| Generalisability | 21 | Discuss the generalisability (external validity) of the study results |  | Discussion paragraphs 6 |
| Other information | |  | | |
| Funding | 22 | Give the source of funding and the role of the funders for the present study and, if applicable, for the original study on which the present article is based |  | Funding |

*Give information separately for cases and controls in case-control studies and, if applicable, for exposed and unexposed groups in cohort and cross-sectional studies.

**Note:** An Explanation and Elaboration article discusses each checklist item and gives methodological background and published examples of transparent reporting. The STROBE checklist is best used in conjunction with this article (freely available on the Web sites of PLoS Medicine at http://www.plosmedicine.org/, Annals of Internal Medicine at http://www.annals.org/, and Epidemiology at http://www.epidem.com/). Information on the STROBE Initiative is available at www.strobe-statement.org.

**Supplementary table 2. Characteristic of the study cohort at baseline**

| Characteristic | Value(n = 7157) |
| --- | --- |
| Age, Mean ± SD | 57.3 ± 8.0 |
| Gender, n (%) |  |
| Female | 3434 (48.0) |
| Male | 3723 (52.0) |
| Education level, n (%) |  |
| High school and above | 989 (13.8) |
| Middle school | 1797 (25.1) |
| Primary school | 1763 (24.6) |
| Illiterate | 2608 (36.4) |
| Marital status, n (%) |  |
| Married | 6553 (91.6) |
| Single | 604 ( 8.4) |
| Residence, n (%) |  |
| Rural | 4449 (62.2) |
| Urban | 2708 (37.8) |
| Smoking status, n (%) |  |
| No | 3763 (52.6) |
| Yes | 3394 (47.4) |
| Drinking.status, n (%) |  |
| No | 3739 (52.2) |
| Yes | 3418 (47.8) |
| BMI, n (%) |  |
| Normal | 4745 (66.3) |
| Obesity | 338 ( 4.7) |
| Overweight | 1754 (24.5) |
| Underweight | 320 ( 4.5) |
| Health.status, n (%) |  |
| Fair | 4541 (63.4) |
| Good | 1523 (21.3) |
| Poor | 1093 (15.3) |
| Physical activity levels |  |
| Low-intensity physical activity | 2219 (31.0) |
| Moderate-intensity physical activity | 995 (13.9) |
| High-intensity physical activity | 3943 (55.1) |
| Depression |  |
| No | 4806 (67.2) |
| Yes | 2351 (32.8) |
| Sleep duration in the wave 1, mean ± SD | 7.0 ± 2.0 |
| Sleep duration in the wave 2, mean ± SD | 6.8 ± 1.9 |
| Sleep duration in the wave 3, mean ± SD | 7.0 ± 2.1 |
| Abbreviations: BMI, body mass index (calculated as weight in kilograms divided by height insquaremeters). | |

**Supplementary table 3. Fit statistics for sleep duration group trajectories**

| Fit statistic | Number of classes | | | | | |
| --- | --- | --- | --- | --- | --- | --- |
|  | 1 | 2 | 3 | 4 | 5 | 6 |
| BIC* | 62467.48 | 59565.34 | 58902.65 | 59004.69 | 58940.77 | 58737.31 |
| AIC* | 62451.48 | 59501.35 | 58798.66 | 58884.70 | 58780.79 | 58561.33 |
| Class proportion | Class 1, 100% | Class 1, 47.74% | Class 1, 34.98% | Class 1, 31.08% | Class 1, 28.13% | Class 1, 18.49% |
|  |  | Class 2, 52.26% | Class 2, 32.67% | Class 2, 25.37% | Class 2, 20.25% | Class 2, 23.60% |
|  |  |  | Class 3, 32.35% | Class 3, 18.87% | Class 3, 14.68% | Class 3, 15.17% |
|  |  |  |  | Class 4, 24.68% | Class 4, 18.47% | Class 4, 14.48% |
|  |  |  |  |  | Class 5, 18.47% | Class 5, 9.59% |
|  |  |  |  |  |  | Class 6, 18.67% |
| APP |  | Class 1, 0.99 | Class 1, 0.99 | Class 1, 0.99 | Class 1, 0.98 | Class 1, 0.98 |
|  |  | Class 2, 0.99 | Class 2, 0.99 | Class 2, 0.99 | Class 2, 0.98 | Class 2, 0.98 |
|  |  |  | Class 3, 0.99 | Class 3, 0.98 | Class 3, 0.98 | Class 3, 0.98 |
|  |  |  |  | Class 4, 0.99 | Class 4, 0.98 | Class 4, 0.99 |
|  |  |  |  |  | Class 5, 0.98 | Class 5, 0.98 |
|  |  |  |  |  |  | Class 6, 0.98 |
| Abbreviations: AIC Akaike’s information criterion, BIC Bayesian information criteria, APP average posterior probabilities | | | | | | |

**Supplementary table 4. The final three-group trajectory model of sleep duration**

**Supplementary table 5. Average duration of sleep in 24 hours, nighttime sleep duration, and daytime sleep in the different sleep duration trajectory groups.**

| Variables | Sleep duration trajectory | | | | |
| --- | --- | --- | --- | --- | --- |
|  | Tota | Persistently long | Persistently moderate | Persistently short | p value |
|  | n = 7157 | n = 2504 | n = 2338 | n = 2315 |  |
| 24-hour sleep duration in 2011 | 7.0 ± 2.0 | 8.6 ± 1.6 | 7.0 ± 0.9 | 5.3 ± 1.8 | < 0.001 |
| 24-hour sleep duration in 2013 | 6.8 ± 1.9 | 8.4 ± 1.6 | 7.0 ± 1.0 | 5.1 ± 1.7 | < 0.001 |
| 24-hour sleep duration in 2015 | 7.0 ± 2.1 | 8.8 ± 1.7 | 7.1 ± 1.0 | 5.2 ± 1.8 | < 0.001 |
| Nighttime sleep duration in 2011 | 6.4 ± 1.8 | 7.7 ± 1.4 | 6.6 ± 1.0 | 5.0 ± 1.7 | < 0.001 |
| Nighttime sleep duration in 2013 | 6.2 ± 1.7 | 7.4 ± 1.4 | 6.4 ± 1.0 | 4.7 ± 1.6 | < 0.001 |
| Nighttime sleep duration in 2015 | 6.4 ± 1.9 | 7.8 ± 1.5 | 6.5 ± 1.0 | 4.8 ± 1.7 | < 0.001 |
| Daytime sleep duration in 2011 | 32.7 ± 42.4 | 51.1 ± 48.9 | 28.4 ± 37.2 | 18.9 ± 33.1 | < 0.001 |
| Daytime sleep duration in 2013 | 38.5 ± 45.1 | 57.2 ± 50.3 | 35.0 ± 40.6 | 23.4 ± 36.8 | < 0.001 |
| Daytime sleep duration in 2015 | 39.8 ± 45.0 | 59.7 ± 50.0 | 35.8 ± 40.4 | 23.9 ± 36.2 | < 0.001 |

| Trajectory group | Parameter | Maximum likelihood estimates | | | |
| --- | --- | --- | --- | --- | --- |
|  |  | Est | SE | z value | p value |
| Class 1: Persistently long(n = 2504, 34.98%) | Intercept | 5.73×10 ^−3^ | 1.06×10 ^−2^ | 0.541 | 0.59 |
|  | Linear (age) | 2..16×10 ^−3^ | 1.42×10 ^−3^ | 1.53 | 0.13 |
|  | Quadratic (age^2^ ) | -9.41×10 ^−5^ | 4.16×10 ^−5^ | -2.26 | 0.02 |
| Class 2: Persistently moderate(n = 2338, 32.67%) | Intercept | 7.79×10 ^−1^ | 1.85×10 ^−2^ | 42.14 | < 0.001 |
|  | Linear (age) | -4.16×10 ^−4^ | 2.36×10 ^−3^ | -0.18 | 0.86 |
|  | Quadratic (age^2^ ) | 1.86×10 ^−4^ | 6.66×10 ^−5^ | 2.82 | 0.005 |
| Class 3: Persistently short(n = 2315, 32.35%) | Intercept | -7.94×10 ^−1^ | 1.26×10 ^−2^ | -62.86 | < 0.001 |
|  | Linear (age) | -4.48×10 ^−3^ | 7.12×10 ^−4^ | 6.29 | < 0.001 |
| Abbreviations: Est. parameter estimate, SE standard error of parameter estimate | | | | | |
